# Connection between maternal suicide attempt and chronic morbidity in children

**DOI:** 10.1017/S0033291722002094

**Published:** 2023-08

**Authors:** Nathalie Auger, Nancy Low, Aimina Ayoub, Jungmin Chang, Thuy Mai Luu

**Affiliations:** 1 University of Montreal Hospital Research Centre, Montreal, Quebec, Canada; 2 Institut national de santé publique du Québec, Montreal, Quebec, Canada; 3 Department of Social and Preventive Medicine, School of Public Health, University of Montreal, Montreal, Quebec, Canada; 4 Department of Epidemiology, Biostatistics, and Occupational Health, McGill University, Montreal, Quebec, Canada; 5 Department of Psychiatry, McGill University, Montreal, Quebec, Canada; 6 Department of Pediatrics, Sainte-Justine Hospital Research Centre, University of Montreal, Montreal, Quebec, Canada

**Keywords:** Allergy and immunology, attempted suicide, child, communicable diseases, dental caries, hospitalization, self-injurious behavior, wounds and injuries

## Abstract

**Background:**

Maternal suicide attempts are associated with adverse psychosocial outcomes in children, but the association with chronic morbidity is poorly understood. We examined the relationship between maternal suicide attempt and risk of hospitalization for potentially preventable conditions in offspring.

**Methods:**

We analyzed a longitudinal cohort of 1 032 210 children born in Quebec, Canada between 2006 and 2019. The main exposure measure was maternal suicide attempt before or during pregnancy. Outcomes included child hospitalizations for potentially preventable conditions, including infectious diseases, dental caries, atopy, and injury up to 14 years after birth. We used adjusted Cox proportional hazards regression models to estimate hazard ratios (HR) and 95% confidence intervals (CI) for the association of maternal suicide attempt with risk of hospitalization for these outcomes.

**Results:**

Compared with no suicide attempt, children whose mothers attempted suicide had an increased risk of hospitalization for infectious diseases (HR 1.11, 95% CI 1.06–1.16), dental caries (HR 1.31, 95% CI 1.15–1.48), and injury (HR 1.16, 95% CI 1.03–1.31). Risk of hospitalization for any of these outcomes was greater if mothers attempted suicide by hanging (HR 1.46, 95% CI 1.22–1.75), had their first attempt between the age of 25 and 34 years (HR 1.27, 95% CI 1.13–1.42), and had 3 or more attempts (HR 1.56, 95% CI 1.27–1.91). Maternal suicide attempts were more strongly associated with child hospitalization before 10 years of age.

**Conclusions:**

Children whose mothers have a history of suicide attempt have an elevated risk of hospitalization for potentially preventable conditions.

## Introduction

Suicide attempts are common in adolescent girls and women of reproductive age, but the health implications for their future children are not well characterized. In the United States, more than 800 000 women had suicide attempts between 2015 and 2019, corresponding to nearly 0.6% of the population (Ivey-Stephenson et al., 2022). Many suicide attempts occur before 25 years of age, when women are of reproductive age and considering pregnancy (Baca-Garcia, Perez-Rodriguez, Mann, & Oquendo, 2008). Women with a history of suicide attempt may have psychosocial or mental health disorders that affect caregiving and the risk of morbidity in children (Howard & Khalifeh, 2020). Studies have shown that women who attempt suicide are more likely to have children who exhibit suicidal behavior and have lower educational attainment (Geulayov, Metcalfe, & Gunnell, 2016; Geulayov, Metcalfe, Heron, Kidger, & Gunnell, 2014). Yet, efforts to determine how maternal suicide attempts relate to other aspects of child health are lacking.

The possibility that maternal suicide attempts before childbirth may be associated with offspring health has not been studied. A longitudinal study of 704 infants found that maternal suicidal ideation during or soon after pregnancy was associated with 45% greater odds of all-cause hospitalization during the first year of life (Crandall, Sridharan, & Schermer, 2010). However, suicide attempts were not examined. The only study that addressed suicide attempts found that infants whose mothers attempted suicide during pregnancy had 20% greater risk of preterm birth, low birth weight, and cesarean delivery (Gandhi et al., 2006). No study has assessed if maternal suicide attempts before pregnancy could predict future child outcomes. These data are needed to improve the management of women with suicidal behavior and prevent child morbidity. We studied the extent to which maternal suicide attempts before childbirth were associated with hospital morbidity in children between birth and 14 years of age.

## Methods

### Study population

This retrospective cohort study included 1 032 210 children born between 2006 and 2019 in Quebec, Canada. We retrieved data for children and their mothers from the Maintenance and Use of Data for the Study of Hospital Clientele repository, which contains all hospital discharge abstracts in Quebec, Canada (Ministry of Health and Social Services, 2017). The charts of each child are linked with their mother. The data include information on all medical and psychiatric diagnoses during admission, and allowed us to identify maternal suicide attempts as far back as 1989.

We followed the children from birth to the end of the study on 31 March 2020, for a maximum of 14 years of follow-up. Children who died at birth and had no follow-up data were excluded from the analysis. We had complete data on 8672 children of mothers with suicide attempts and 1 023 538 children of mothers with no attempt.

### Measures

#### Maternal suicide attempt

The exposure of interest was any maternal history of suicide attempt before or during pregnancy. We identified women who were hospitalized for suicide attempts using diagnostic codes for intentional self-harm in the 9th and 10th revisions of the International Classification of Diseases (online Supplementary Table S1). We classified suicide attempts by age (<15, 15–17, 18–24, 25–34, ⩾35 years) and method (poisoning, hanging, cutting or piercing, other) at first attempt. The category for other methods included drowning, firearm, smoke, fire, heat, jumping from a high place or before a moving object, motor vehicle accident, caustic substances, electrocution, and unspecified means. We also determined the total number of attempts before pregnancy (1, 2, 3 or more). We used the method and number of suicide attempts as indicators of severity.

#### Child hospitalization

The main outcome measure included child hospitalizations for infectious diseases (respiratory, otitis media, gastroenteritis, encephalitis and meningitis, septicemia, skin, vaccine-preventable infections, other), dental caries requiring in-hospital treatment, atopic conditions (asthma, anaphylaxis, dermatitis, other), and injury (fracture, wounds and dislocations, amputation, crush, and nerve injuries, concussion, burns, poisoning, foreign body, maltreatment-related injuries) (online Supplementary Table S1). We selected these outcomes because they are common reasons for hospitalization in childhood that are potentially preventable.

We additionally identified children who were hospitalized for appendicitis or cancer. We used these two outcomes as negative controls, as they are less easily preventable or affected by a maternal history of suicide attempt.

#### Covariates

Maternal confounders included age at childbirth (<25, 25–34, ⩾35 years), parity (0, 1, ⩾2 previous deliveries), history of mental disorders defined as schizophrenia, mood, anxiety, stress, and personality disorders before or during pregnancy (yes, no), child sex (female, male), preterm birth defined as <37 weeks of gestation (yes, no), socioeconomic disadvantage (yes, no, unspecified), place of residence (rural, urban, unspecified), and year of childbirth (2006–2010, 2011–2014, 2015–2019). We captured mental disorders using hospital discharge records before pregnancy and prenatal obstetric charts available at the delivery hospitalization. Prenatal obstetric charts include information on mental illness anytime during pregnancy. Socioeconomically disadvantaged patients included children in the most materially deprived quintile of the population based on a neighborhood-level index of education, employment, and income (Auger, Low, Lee, Lo, & Nicolau, 2020).

### Statistical analysis

We calculated hospitalization rates for each outcome and the cumulative incidence at 14 years of age. We used Cox proportional hazards regression models to estimate hazard ratios (HR) and 95% confidence intervals (CI) for the association of maternal suicide attempt with child hospitalization. We computed both unadjusted and adjusted HRs, accounting for maternal age, parity, mental disorders, child sex, preterm birth, socioeconomic disadvantage, place of residence, and year of childbirth. The time scale was expressed in days from birth to the first hospitalization for each outcome, death, or study end. We censored children who were never hospitalized during the study period, accounted for siblings in the same family using robust error estimates, and controlled for death as a competing event using the Fine and Gray method.

In secondary analyses, we assessed risks by method and age at first suicide attempt, as well as total number of attempts. We calculated age-specific risks of hospitalization at age <1, 1–4, 5–9, and ⩾10 years. In sensitivity analyses, we examined the association of maternal suicide attempts with mental illness hospitalization after birth, as well as the association of maternal mental illness after birth with child hospitalization. Finally, we accounted for suicide attempts after childbirth and for the combined presence of suicide attempts and mental illness.

We performed the analysis in SAS v9.4 (SAS Institute Inc., Cary, NC). We utilized an anonymized dataset and received an ethics waiver from the institutional review board of the University of Montreal Hospital Centre.

## Results

The cohort comprised 1 032 210 children born between 2006 and 2019, including 8672 (0.8%) whose mothers attempted suicide any time before the birth of their child (Table 1). 99.0% of attempts occurred before pregnancy. A total of 220 519 children were hospitalized for any study outcome during 6 409 346 person-years of follow-up, including 2757 children (1.3%) born to mothers with a history of suicide attempt. Compared with no history of suicide attempt, women who attempted suicide were more likely to have a mental disorder, be under 25 years, multiparous, socioeconomically disadvantaged, and live in a rural area when their child was born.

**Table 1.** Child characteristics according to maternal history of suicide attempt

	No. children (%)	
	Maternal suicide attempt	No suicide attempt
Maternal age at childbirth, years		
<25	2262 (26.1)	156 241 (15.3)
25–34	5140 (59.3)	681 281 (66.6)
⩾35	1270 (14.6)	186 016 (18.2)
Parity		
0	3558 (41.0)	500 663 (48.9)
1	2700 (31.1)	357 018 (34.9)
⩾2	2414 (27.8)	165 857 (16.2)
Mental disorder		
Any mental disorder	6536 (75.4)	41 970 (4.1)
Schizophrenia	280 (3.2)	1844 (0.2)
Mood disorder	2456 (28.3)	12 985 (1.3)
Anxiety or stress disorder	5178 (59.7)	30 659 (3.0)
Personality disorder	3095 (35.7)	9382 (0.9)
No mental disorder	2136 (24.6)	981 568 (95.9)
Child sex		
Female	4268 (49.2)	498 330 (48.7)
Male	4404 (50.8)	525 208 (51.3)
Preterm birth		
Yes	864 (10.0)	66 603 (6.5)
No	7808 (90.0)	956 935 (93.5)
Socioeconomic disadvantage		
Yes	2659 (30.7)	204 137 (19.9)
No	5692 (65.6)	778 328 (76.0)
Place of residence		
Rural	2566 (29.6)	185 881 (18.2)
Urban	6008 (69.3)	820 334 (80.2)
Year of childbirth		
2006–2010	3096 (35.7)	384 529 (37.6)
2011–2014	2712 (31.3)	322 147 (31.5)
2015–2019	2864 (33.0)	316 862 (31.0)
Total	8672 (100.0)	1 023 538 (100.0)

Maternal suicide attempt was associated with an increased risk of child hospitalization before 14 years of age (Table 2). In adjusted models, children of women with suicide attempts had 1.12 times the risk of hospitalization for any study outcome (95% CI 1.07–1.16) compared with no attempt. Children whose mothers attempted suicide had 1.11 times the risk of hospitalization for infectious diseases (95% CI 1.06–1.16), 1.31 times the risk for dental caries (95% CI 1.15–1.48), and 1.16 times the risk for injury (95% CI 1.03–1.31). Among specific outcomes, associations were strongest for septicemia (HR 1.60, 95% CI 1.11–2.29), skin infection (HR 1.46, 95% CI 1.21–1.78), advanced dental caries (HR 1.46, 95% CI 1.21–1.77), and concussion (HR 1.67, 95% CI 1.07–2.60). Maternal suicide attempt was not associated with atopic conditions, appendicitis, and cancer morbidity.

**Table 2.** Association of maternal suicide attempt with type of child hospitalization

	Maternal suicide attempt		No suicide attempt		Hazard ratio (95% CI)^a^	
	No. children	Rate per 10 000 person-years	No. children	Rate per 10 000 person-years	Unadjusted	Adjusted^b^
Infectious disease	2275	465.7	180 036	276.5	1.60 (1.53–1.67)	1.11 (1.06–1.16)
Respiratory	1542	290.8	116 191	168.6	1.65 (1.56–1.74)	1.11 (1.05–1.17)
Otitis media	962	168.1	77 767	108.6	1.51 (1.41–1.61)	1.03 (0.96–1.11)
Gastroenteritis	328	54.1	24 647	33.1	1.60 (1.43–1.80)	1.00 (0.89–1.13)
Encephalitis, meningitis	33	5.3	3213	4.2	1.22 (0.87–1.72)	0.93 (0.65–1.34)
Septicemia	37	5.9	2184	2.9	2.02 (1.45–2.82)	1.60 (1.11–2.29)
Skin	130	20.9	8131	10.7	1.93 (1.62–2.30)	1.46 (1.21–1.78)
Vaccine preventable	88	14.2	6836	9.0	1.54 (1.24–1.90)	1.07 (0.85–1.35)
Other	64	10.3	4364	5.7	1.73 (1.36–2.22)	1.12 (0.86–1.46)
Dental caries	400	65.5	19 786	26.3	2.52 (2.24–2.82)	1.31 (1.15–1.48)
Mild	369	60.3	17 049	22.6	2.69 (2.38–3.03)	1.33 (1.17–1.52)
Advanced	169	27.2	6446	8.5	3.22 (2.73–3.80)	1.46 (1.21–1.77)
Atopic condition	431	71.3	31 897	42.9	1.64 (1.48–1.81)	1.07 (0.97–1.19)
Asthma	321	52.6	22 864	30.5	1.70 (1.52–1.91)	1.08 (0.95–1.22)
Anaphylaxis	24	3.8	2005	2.6	1.44 (0.97–2.15)	1.09 (0.71–1.69)
Dermatitis	81	13.0	6598	8.7	1.47 (1.18–1.84)	1.05 (0.82–1.34)
Other	60	9.6	4494	5.9	1.61 (1.25–2.07)	1.10 (0.84–1.45)
Injury	336	55.1	24 364	32.5	1.69 (1.51–1.89)	1.16 (1.03–1.31)
Fracture	119	19.1	11 688	15.5	1.24 (1.03–1.49)	1.03 (0.84–1.25)
Wounds and dislocations	78	12.5	5029	6.6	1.88 (1.50–2.35)	1.13 (0.88–1.44)
Amputation, crush, and nerve injuries	87	13.9	5156	6.8	2.04 (1.65–2.52)	1.27 (1.00–1.60)
Concussion	25	4.0	970	1.3	3.11 (2.09–4.62)	1.67 (1.07–2.60)
Burns, poisoning, foreign body	95	15.3	5753	7.6	1.99 (1.63–2.44)	1.23 (0.99–1.54)
Maltreatment	27	4.3	805	1.1	3.99 (2.72–5.85)	1.20 (0.78–1.83)
Appendicitis	53	8.5	4694	6.2	1.40 (1.07–1.84)	1.31 (0.98–1.75)
Cancer	17	2.7	1649	2.2	1.24 (0.77–2.00)	1.21 (0.72–2.02)
Any outcome	2757	608.9	217 762	348.7	1.63 (1.57–1.69)	1.12 (1.07–1.16)

a Hazard ratio for maternal suicide attempt relative to no attempt.

b Adjusted for maternal age, parity, mental illness, child sex, preterm birth, socioeconomic disadvantage, place of residence, and year of childbirth.

Risk of child hospitalization varied by method, age, and total number of maternal suicide attempts (Table 3). In adjusted models, attempts by hanging were associated with hospitalization for infectious diseases (HR 1.29, 95% CI 1.08–1.55) and dental caries (HR 2.66, 95% CI 1.72–4.11). Associations were somewhat weaker for attempts by poisoning. Attempts between 18 and 34 years were also associated with these outcomes. Having three or more suicide attempts was strongly associated with the risk of any child hospitalization (HR 1.56, 95% CI 1.27–1.91), especially injuries (HR 2.09, 95% CI 1.21–3.61) and infectious diseases (HR 1.58, 95% CI 1.28–1.95).

**Table 3.** Characteristics of maternal suicide attempts and risk of child hospitalization

	Hazard ratio (95% CI)^a^			
	Infectious disease	Dental caries	Injury	Any outcome
Method at first attempt				
Poisoning	1.11 (1.05–1.17)	1.26 (1.09–1.44)	1.10 (0.96–1.26)	1.09 (1.04–1.14)
Hanging	1.29 (1.08–1.55)	2.66 (1.72–4.11)	1.69 (1.07–2.66)	1.46 (1.22–1.75)
Cutting or piercing	0.95 (0.80–1.13)	1.40 (0.99–1.98)	1.15 (0.78–1.70)	1.03 (0.88–1.20)
Other	1.24 (1.01–1.52)	0.66 (0.35–1.24)	1.95 (1.23–3.09)	1.18 (0.96–1.44)
No attempt	Reference	Reference	Reference	Reference
Age at first attempt, years				
<15	1.11 (1.01–1.22)	1.21 (0.92–1.60)	1.16 (0.91–1.47)	1.11 (1.02–1.22)
15–17	1.02 (0.94–1.10)	1.09 (0.89–1.35)	1.15 (0.94–1.40)	1.02 (0.95–1.10)
18–24	1.15 (1.07–1.25)	1.48 (1.22–1.79)	1.20 (0.99–1.46)	1.14 (1.06–1.23)
25–34	1.28 (1.14–1.44)	1.75 (1.31–2.32)	1.06 (0.75–1.50)	1.27 (1.13–1.42)
⩾35	0.73 (0.43–1.23)	1.23 (0.40–3.80)	1.63 (0.60–4.42)	0.66 (0.39–1.13)
No attempt	Reference	Reference	Reference	Reference
Number of attempts				
⩾3	1.58 (1.28–1.95)	1.07 (0.57–2.02)	2.09 (1.21–3.61)	1.56 (1.27–1.91)
2	1.10 (0.96–1.26)	1.69 (1.25–2.29)	1.27 (0.90–1.80)	1.15 (1.02–1.31)
1	1.09 (1.04–1.15)	1.27 (1.11–1.45)	1.12 (0.98–1.27)	1.08 (1.03–1.13)
No attempt	Reference	Reference	Reference	Reference

a Hazard ratio for maternal suicide attempt relative to no attempt, adjusted for maternal age, parity, mental illness, child sex, preterm birth, socioeconomic disadvantage, place of residence, and year of childbirth.

**Fig. 1.**
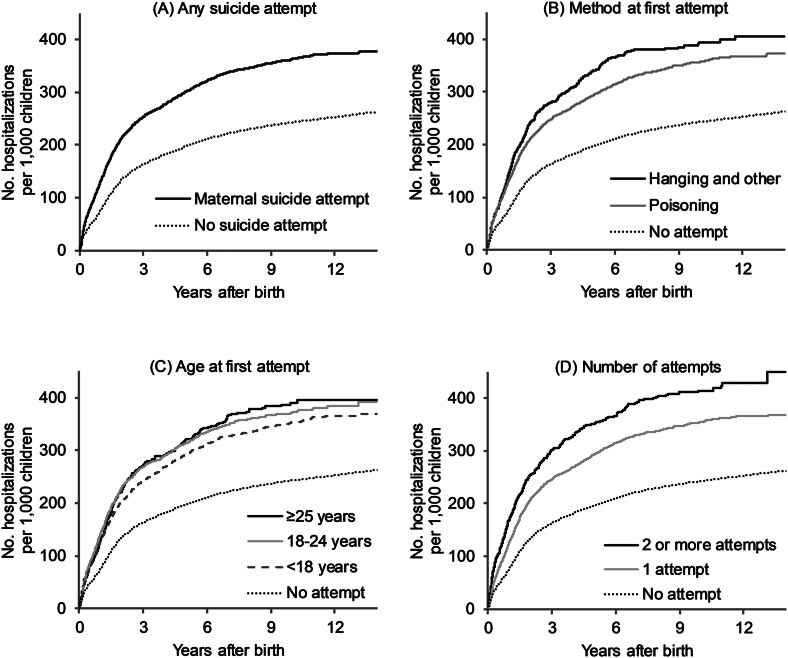
Cumulative incidence of child hospitalization according to type of maternal suicide attempt.

Hospitalization rates reached 380.4 per 1000 at 14 years of age for children with a maternal history of suicide attempt, compared with 263.8 per 1000 for unexposed children (Fig. 1). Child hospitalization rates increased more rapidly in the first six years after birth. This trend was apparent regardless of the method, age, or total number of suicide attempts.

Relative to no suicide attempt, children whose mothers attempted suicide were at greatest risk of hospitalization before 10 years of age (Table 4). In adjusted models, maternal suicide attempts were associated with 1.12 times the risk of hospitalization before 1 year (95% CI 1.05–1.19), 1.11 times the risk between 1 and 4 years (95% CI 1.05–1.18), and 1.18 times the risk between 5 and 9 years (95% CI 1.03–1.34), but there was no association in older children. Associations were similar for suicide attempt by poisoning. Attempts by hanging were associated with hospitalization between 1 and 4 years (HR 1.65, 95% CI 1.30–2.10). Attempts between 18 and 24 years were associated with hospitalization before 5 years, whereas attempts between 25 and 34 years were associated with hospitalization between 1 and 9 years. Children whose mothers attempted suicide 3 times or more had 1.82 times the risk of hospitalization before 1 year (95% CI 1.39–2.37).

**Table 4.** Association of maternal suicide characteristics with age-specific risk of child hospitalization

	Hazard ratio (95% CI)^a^			
	<1 year	1–4 years	5–9 years	⩾10 years
Any suicide attempt	1.12 (1.05–1.19)	1.11 (1.05–1.18)	1.18 (1.03–1.34)	0.87 (0.56–1.36)
Method at first attempt				
Poisoning	1.12 (1.04–1.20)	1.09 (1.02–1.16)	1.20 (1.05–1.38)	0.90 (0.57–1.43)
Hanging	1.21 (0.94–1.56)	1.65 (1.30–2.10)	1.12 (0.48–2.61)	2.34 (0.32–17.2)
Cutting or piercing	0.96 (0.76–1.22)	1.07 (0.87–1.31)	0.94 (0.56–1.59)	0.63 (0.09–4.35)
Other	1.24 (0.93–1.65)	1.25 (0.94–1.67)	1.06 (0.58–1.95)	–
No attempt	Reference	Reference	Reference	Reference
Age at first attempt, years				
<15	1.14 (0.99–1.30)	1.14 (1.01–1.29)	1.07 (0.81–1.42)	0.55 (0.17–1.73)
15–17	1.04 (0.93–1.16)	1.02 (0.92–1.13)	1.19 (0.97–1.45)	1.09 (0.57–2.06)
18–24	1.18 (1.06–1.32)	1.13 (1.02–1.25)	1.12 (0.89–1.40)	1.10 (0.54–2.23)
25–34	1.16 (0.98–1.38)	1.33 (1.13–1.55)	1.48 (1.04–2.12)	0.34 (0.05–2.48)
⩾35	0.80 (0.39–1.62)	0.74 (0.38–1.44)	1.75 (0.66–4.66)	–
No attempt	Reference	Reference	Reference	Reference
Number of attempts				
⩾3	1.82 (1.39–2.37)	1.29 (0.96–1.73)	0.64 (0.24–1.70)	1.34 (0.18–9.76)
2	1.20 (1.00–1.44)	1.11 (0.93–1.33)	1.31 (0.91–1.87)	1.34 (0.43–4.22)
1	1.08 (1.01–1.16)	1.11 (1.04–1.18)	1.18 (1.03–1.35)	0.81 (0.50–1.32)
No attempt	Reference	Reference	Reference	Reference

a Hazard ratio for maternal suicide attempt relative to no attempt, adjusted for maternal age, parity, mental illness, child sex, preterm birth, socioeconomic disadvantage, place of residence, and year of childbirth.

In sensitivity analyses, maternal attempts before or during pregnancy were associated with 1.18 times the risk of maternal mental illness hospitalization after birth (95% CI 1.11–1.26) (online Supplementary Figure S1). Maternal mental illness hospitalization after birth was in turn associated with 1.41 times the risk of child hospitalization (95% CI 1.38–1.44). Women whose first suicide attempt was after childbirth had a greater risk of child hospitalization (HR 1.49, 95% CI 1.41–1.58) than women with a repeat attempt after birth (HR 1.23, 95% CI 1.06–1.44) or attempt before birth only (HR 1.12, 95% CI 1.07–1.17), compared with no attempt (online Supplementary Table S2). Risk of hospitalization in children whose mothers had both suicide attempts and mental illness was similar to the risk for mothers with suicide attempts only or mental illness only, compared with neither exposure.

## Discussion

In this cohort of 1 million children, maternal history of suicide attempt before or during pregnancy was associated with a greater risk of child hospitalization for potentially preventable conditions. Maternal suicide attempts were associated with childhood hospitalizations for infectious diseases, dental caries, and injuries. Risk of hospitalization was particularly elevated for children whose mothers attempted suicide between 25 and 34 years of age, by hanging, and had repeated attempts. There was however no association with cancer and appendicitis, conditions that are less easily preventable. The findings suggest that maternal suicide attempts may be a risk factor for conditions that are potentially avoidable in childhood, and highlight the need for early screening and support of new mothers with a history of suicidal behavior.

Very little is known on the relationship between maternal suicidal behavior and offspring health. The only longitudinal data available are limited to a cohort of 704 children exposed to maternal suicidal ideation during or soon after birth (Crandall et al., 2010). Suicidal ideation was associated with 45% greater odds of all-cause hospitalization during the first year of infancy; however, the cohort included mothers with depression only (Crandall et al., 2010). The investigators were able to demonstrate that there was no association with injuries, but outcomes after 1 year of age were not examined. Other researchers have shown that women with suicide attempts are at greater risk of preterm birth, low birth weight, cesarean delivery, and stillbirth (Gandhi et al., 2006; Zhong et al., 2018). Our study suggests that maternal suicide attempts may be associated with an increased risk of admission throughout childhood, not only the first year of life.

A larger number of studies have assessed the association of maternal suicidal behavior with child mental and cognitive development (Cerel, Fristad, Weller, & Weller, 1999; Geulayov et al., 2014, 2016; Mebrahtu et al., 2020; Sheftall et al., 2020). In a study of 3496 mothers, self-reported suicide attempts were associated with a 3-fold increased risk of suicide attempt and suicidal ideation in offspring at adolescence (Geulayov et al., 2014). A study of 9721 children from the UK found that maternal suicide attempts were associated with lower educational attainment at 14 years (Geulayov et al., 2016). In an analysis of 26 children and 332 controls aged 5–17 years, children who lost their parents by suicide tended to have more depressive symptoms compared with children whose parents died from other causes (Cerel et al., 1999). Some data suggest that maternal suicidal behavior is associated with poor cognitive and emotional development, including sadness, discomfort, and less resilience early in childhood (Mebrahtu et al., 2020; Sheftall et al., 2020). These studies all support the possibility that maternal suicidal behavior may be a determinant of physical morbidity in childhood.

The relationship between suicide attempt and child morbidity may be mediated by maternal mental illness, a known risk factor for adverse child outcomes (Auger et al., 2020, 2021; Giallo et al., 2015; Nevriana et al., 2020; O'Donnell et al., 2015). Previous longitudinal studies have shown that maternal mental disorders are associated with pediatric infections (Auger et al., 2021), dental caries (Auger et al., 2020), and injuries (Nevriana et al., 2020). Australian cohorts have linked maternal mental disorders with more than 2 times the risk of child asthma (Giallo et al., 2015) and maltreatment (O'Donnell et al., 2015). Although the associations in our data were attenuated after adjusting for mental disorders, maternal suicide attempts remained associated with a 10–30% greater risk of hospitalization for infectious diseases, dental caries, and injury overall. For this reason, mental disorders likely explain only part of the association between suicide attempt and child morbidity.

Parental stress, financial hardship, and low social support may be additional pathways (Fang et al., 2014; Milam et al., 2008; Sidebotham & Heron, 2006). All of these factors have been linked with child morbidity in past research (Fang et al., 2014; Milam et al., 2008; Sidebotham & Heron, 2006). In a cohort study of 2888 children between 5 and 7 years from the U.S., parental stress was associated with 1.2 times the risk of child asthma symptoms, especially among boys (Milam et al., 2008). A study of 98 385 children under 18 years in China found that financially disadvantaged children had 1.7 times the risk of fall injuries compared with financially advantaged children (Fang et al., 2014). In the UK, children whose parents had limited social networks were 2 times more likely to experience maltreatment than children with more diverse social networks (Sidebotham & Heron, 2006). The cognitive burden of parental stress, financial strain, and limited social support may impair parenting skills and mother-child interactions, which can disrupt healthy physical development and delay ambulatory care.

Risk of child morbidity may vary depending on the timing and severity of maternal suicide attempts. In a study of 319 women with suicide attempts during pregnancy, hanging and other violent means were associated with 3.6 times the odds of fetal death compared with self-poisoning or cutting (Shigemi, Ishimaru, Matsui, Fushimi, & Yasunaga, 2021). Some of the association may be due to a direct effect of the suicide attempt on the fetus. Nevertheless, our study suggests that suicide attempts by hanging before pregnancy are also associated with adverse childhood outcomes. Risk is also elevated for women with repeated attempts. Women with more serious suicide attempts are more likely to have mental, interpersonal, or financial challenges that affect the ability to care for children (Beautrais, Joyce, & Mulder, 1997; Forman, Berk, Henriques, Brown, & Beck, 2004).

Moreover, maternal suicide attempts in adulthood were associated with a greater risk of child hospitalization than attempts in adolescence, especially for infection and dental caries. Adults tend to have different reasons for suicide attempt than adolescents. Adults are more likely to attempt suicide for job-related or economic difficulties (Lee et al., 2019). Adults also tend to have more serious suicide attempts with stronger intention to die (Lee et al., 2019), reflecting more complex psychosocial problems. In a Swedish study of 53 843 suicide attempts and 538 393 controls, women aged ⩾20 years were more likely to use violent means and have repeated attempts compared with adolescent girls aged 10–19 years (Tidemalm et al., 2015). Mental and alcohol-related disorders are also more frequent in adults (Ong et al., 2021; Tidemalm et al., 2015). Compared with adolescents, adults may be less likely to receive counseling and psychotherapy, or have social support following a suicide attempt (Cheung & Dewa, 2007). Overall, our findings suggest that women who attempt suicide after 18 years should be followed more closely.

In this study, children whose mothers attempted suicide were at the greatest risk of hospitalization between birth and 10 years of age. Studies have shown that maternal mental illness is also associated with a greater risk of morbidity before 10 years, including dental caries (Auger et al., 2020) and pediatric infectious disease hospitalization (Auger et al., 2021). The findings highlight the potential for maternal mental illness to impede a mother's ability to care for young children, especially if there is a history of suicidal behavior. Younger children depend more on their parents and generally require more supervision. Children become more independent as they grow and may be less impacted by their mothers' mental health, which could explain the lack of association between maternal suicide attempt and hospitalization at older ages.

### Limitations

This study has limitations that should be considered. We used hospital discharge records and were not able to identify maternal suicide attempts that did not require hospitalization. Exposure misclassification is likely nondifferential, although the associations may be underestimated. We could not differentiate between confirmed and suspected suicide attempts. As suicide attempts during pregnancy were rare, we could not examine this exposure separately. We had data on age, method, and number of suicide attempts, but lacked information on circumstances surrounding the attempt, including family context and use of mental health services. We could not determine whether mothers received psychotherapy or medical treatment after a suicide attempt. We captured child outcomes that were serious enough to require in-hospital treatment. Future studies should explore associations with less severe conditions. Residual confounding may be present as we could not account for ethnicity, marital status, social support, paternal history of suicide attempt, domestic violence, adverse childhood events, or maternal behaviors such as alcohol use and smoking. Mental disorders may be underreported. The long timespan of the study increases the possibility of confounding. Generalizability of the findings to populations in different settings is to be determined.

## Conclusions

The findings of this population-based cohort study suggest that maternal suicide attempts may predispose offspring to potentially preventable childhood morbidity, including infectious diseases, dental caries, and injury hospitalization the first 10 years of life. Children whose mothers attempt suicide by hanging, are adults at the time of the attempt, or have repeated attempts, are at the greatest risk. The results raise concern in light of increasing rates of suicide attempt and mental health disorders in women of reproductive age (Yard et al., 2021), and support the need for screening, education, and psychosocial support in this population. Ongoing monitoring of women with a history of suicide attempt may help improve health outcomes in children.

## Supplementary material

For supplementary material accompanying this paper visit https://doi.org/10.1017/S0033291722002094. click here to view supplementary material
