# Supplementary material for: Connection between maternal suicide attempt and chronic morbidity in children
Source: Psychol Med. 2022 Jul 15;53(11):5091–8. doi: 10.1017/S0033291722002094 (PMC10476049; doi:10.1017/S0033291722002094)
Supplement: Supplementary file 1 [file S0033291722002094sup001.docx]

**Figure S1** Associations between maternal suicide attempt, onset of maternal mental illness after birth, and child hospitalization^a^


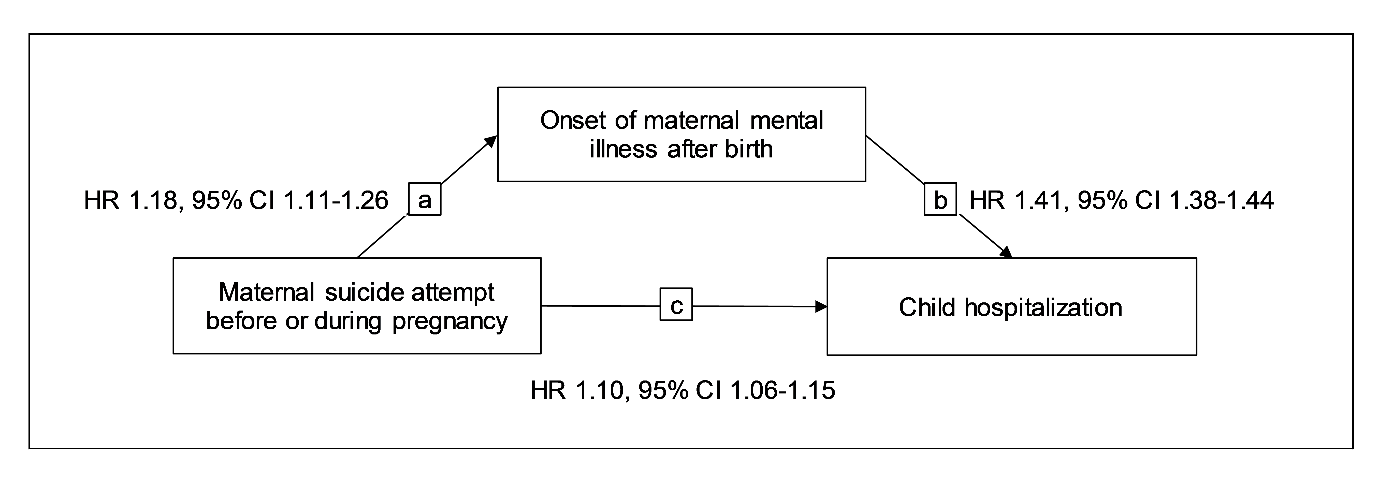
^a^Models a and b are adjusted for maternal age, parity, mental illness, child sex, preterm birth, socioeconomic disadvantage, place of residence, and year of childbirth. Model c is additionally adjusted for the onset of maternal mental illness after birth.**Table S1** Diagnostic codes for suicide attempt and child outcomes

|  | International Classification of Diseases, tenth revision |
| --- | --- |
| Suicide attempt | X60-X84, Y87.0 |
| Method at suicide attempt |  |
| Poisoning | X60-X69 |
| Hanging | X70 |
| Cutting or piercing | X78, X79 |
| Other | X71-X77, X80-X84, Y87.0 |
| Infectious disease |  |
| Respiratory | J00-J06, J09-J18, J20-J22, J30 |
| Otitis media | H66, H67, H92 |
| Gastroenteritis | A00-A09 |
| Encephalitis, meningitis | A83-A87, A92.2, A92.3, B94.1, G00-G05 |
| Septicemia | A40, A41, R57.2 |
| Skin | L00-L08 |
| Vaccine preventable | A08.0, A35-A37, A39, A40.3, A41.3, A49.2, A80, B01, B05, B06, B15, B16, B26, B91, B95.3, B96.3 |
| Other | P23, P35-P39 |
| Dental caries |  |
| Mild | K02 |
| Advanced | K04.0-K04.7, K05.2, K05.3 |
| Atopic condition |  |
| Asthma | J45 |
| Anaphylaxis | T78.0, T78.2-T78.4, T80.5, T88.6, T88.7 |
| Dermatitis | L20, L23, L27 |
| Other | D69.0, J67, J82, K52.2, L23, L50.0, M13.8, M30.1, Z88, Z91.0 |
| Injury |  |
| Fracture | S02, S12, S22, S32, S42, S52, S62, S72, S82, S92, T02.0-T02.7, T08, T10, T12 |
| Wounds and dislocations | S00, S01, S03, S10, S11, S13, S20, S21, S23, S30, S31, S33, S40, S41, S43, S50, S51, S53, S60, S61, S63, S70, S71, S73, S80, S81, S83, S90, S91, S93, T00.0-T00.3, T01.0-T01.3, T03.0-T03.3, T09.0, T09.1, T11.0-T11.2, T13.0-T13.2 |
| Amputation, crush, and nerve injuries | S04-S08, S14-S18, S24-S28, S34-S38, S44-S48, S54-S58, S64-S68, S74-S78, S84-S88, S94-S98, T04.0-T04.3, T05.0-T05.5, T11.3, T11.6, T13.3, T13.6 |
| Concussion | S06.0 |
| Burns, poisoning, foreign body | T15-T19, T20-T32, T36-T50, X40-X49, W44, W45 |
| Maltreatment | T73, T74, X85-X99, Y00-Y09 |
| Appendicitis | K35-K37 |
| Cancer | Codes published in Steliarova-Foucher et al^a^ |

^a^Steliarova-Foucher, E., Stiller, C., Lacour, B., & Kaatsch, P. (2005). International Classification of Childhood Cancer, third edition. *Cancer,* *103*(7), 1457–1467. doi:10.1002/cncr.20910**Table S2** Association of maternal suicide attempt and mental illness with any child hospitalization

|  | Hazard ratio (95% CI)^a^ |
| --- | --- |
| Maternal suicide and mental illness before or during pregnancy |  |
| Suicide attempt and mental illness combined | 1.45 (1.38-1.53) |
| Suicide attempt only | 1.48 (1.36-1.60) |
| Mental illness only | 1.44 (1.41-1.47) |
| No suicide attempt or mental illness | Reference |
| Timing of maternal suicide attempt^b^ |  |
| First attempt after childbirth | 1.49 (1.41-1.58) |
| Repeat attempt after childbirth | 1.23 (1.06-1.44) |
| Attempt before childbirth only | 1.12 (1.07-1.17) |
| No attempt | Reference |

^a^Adjusted for maternal age, parity, child sex, preterm birth, socioeconomic disadvantage, place of residence, and year of childbirth.

^b^Additionally adjusted for maternal mental illness before or during pregnancy.
